# Supplementary material for: Positive Selection Drives cis-regulatory Evolution Across the Threespine Stickleback Y Chromosome
Source: Mol Biol Evol. 2024 Feb 2;41(2):msae020. doi: 10.1093/molbev/msae020 (PMC10899008; doi:10.1093/molbev/msae020)
Supplement: msae020_Supplementary_Data [file msae020_supplementary_data.zip › Supplemental Figures.pdf]

Supplemental Figures

Supplemental Figure 1. Nucleotide diversity (pi) across the sex chromosomes and representative autosome. The Y chromosome exhibits a strong reduction in nucleotide diversity consistent with a lower effective population size, and selection. Estimates of nucleotide diversity were calculated from 12 male individuals from a freshwater population. Nucleotide diversity was averaged across 10,000bp windows for A. Chromosome 18, B. X chromosome, and C. Y chromosome. D. The Y chromosome has lower diversity than both the X chromosome and chromosome 18**.** E. Distinct strata on the X chromosome have varying levels of the nucleotide diversity. A Kruskal Wallis test indicated differences between the groups ($P < 0.001$). A post-Hoc Dunn's test and Bonferroni Correction revealed that the PAR was significantly higher from all three strata ($P < 0.001$). Stratum one was significantly higher than stratum two and three ($P < 0.001$). After removing windows that overlapped the centromeric region, stratum two and three are not significantly different ($P = 0.937$). F. All three strata across the Y chromosome have similar estimates of nucleotide diversity ($P=0.768$, Kruskal Wallis test). The PAR was not analyzed for the Y chromosome as the assembly is missing most of the PAR sequence. The letters above each plot indicate the significant differences between sites within each stratum ($P < 0.001$). Box plots that share a letter assignment are not significantly different from one another ($P > 0.001$).

Supplemental Figure 2. d_N / d_S ratios of coding regions compared to accessible chromatin region divergence. There is no correlation between d_N / d_S and ACR divergence using a Spearman's Rank Correlation using ACRs from liver (A and B) or testis (C and D) tissue. Liver: (**A**) Y divergence: (Stratum one (Blue): R=-0.12 P = 0.230, N = 30, Stratum two (Yellow): R = 0.2, P = 0.430, N=61; Stratum three (Grey): R = 0.11, P = 0.770 N=126. (**B**) X divergence: (Stratum one (Blue): R = -0.080, P = 0.420, N=30; Stratum two (Yellow): R = 0.032, P = 0.9, N = 61; Stratum three (Grey): R = -0.054, P = 0.890, N = 126. Similar results in testis: (**C**) Y divergence: (Stratum one (Blue): R = -0.020, P = 0.650, N=14; Stratum two (Yellow): R = -0.430, P = 0.160, N = 12; Stratum three (Grey): R = -0.140, P = 0.480, N = 17. (**D**) X divergence: (Stratum one (Blue): R = 0.520, P = 0.120, N=14; Stratum two (Yellow): R = -0.360, P = 0.210, N = 12; Stratum three (Grey): R = -0.020, P = 0.670, N = 17.

Supplemental Figure 3. Selective constraint of genes affects correlation between ACR and proximal coding divergence. Y-linked gametologs were classified by their mode of selection based on a McDonald Kreitman Test. Genes were classified as Positive selection: $NI_{Haldane} < -0.25$, Relaxed purifying selection: $-0.25 < NI_{Haldane} < 0.25$, and purifying selection: $NI_{Haldane} > 0.25$. Genes likely evolving under positive selection had Y-linked and X-linked ACR divergence significantly correlated with d_N/d_S (Y chromosome: R=0.92, P=0.002, N=10; X chromosome: R = 0.81, P = 0.03, Spearman's Rank correlation coefficient). Genes under purifying selection and relaxed purifying selection did not show correlations between ACR divergence and DN/DS (Purifying: Y chromosome: R= -0.14, P = 0.30, N=57; X chromosome: R= -0.022, P =0.87, N=57; Relaxed purifying selection: Y chromosome: R= -0.293, P =0.0155, N=35, X chromosome: R= -0.32, P =0.063, N=35, Spearman's Rank Correlation coefficient. Grey areas represent 95% confidence intervals. Outliers with $d_N/d_S > 3$ were removed prior to analysis. When $d_N/d_S > 1$ are removed the relationship for genes under positive selection is still significant (R=0.86, P=0.006).

Supplemental figure 4. Rates of allelic expression for Y gametologs under different modes of selection. Y-linked gametologs were classified by their mode of selection based on a McDonald Kreitman Test. Genes were classified as Positive selection: $NI_{Haldane} < -0.25$, Relaxed constraint selection: $-0.25 < NI_{Haldane} < 0.25$, and purifying selection: $NI_{Haldane} > 0.25$. X/Y gene expression was quantified based on counts from liver and testis RNA-seq data from 3 individuals. Counts were averaged across all three replicates. Rates of X/Y expression are not significantly different for each class (Kruskal-Wallis test).

Supplemental Figure 5. Accessible chromatin region divergence compared to functional states of Y-linked gametologs. ACR divergence was not significantly different among gametologs that contained mutations that potentially impede function (i.e., premature stop codons, frameshifts, deleted nucleotides) or are functional. Y-specific functional mutations were identified by comparing coding sequence between the Y chromosome and the outgroup autosomal coding sequence in the ninespine stickleback. ACRs were assigned to the closest gametolog within 50kb. $P > 0.05$ for all pairwise comparisons for both liver (A and C) and testis (B and D) for regulatory divergence on the X and Y.

Supplemental Figure 6. High concordance of chromatin accessibility around transcription start site of expressed genes. Transcription start sites (TSSs) for all genes were ordered by RNA-seq read depth. ATAC-seq read depth was quantified ±3 kb of TSSs. Similar ATAC-seq read depth was observed between replicates. ATAC-seq depth across replicates is a strong predictor of expression from RNA-seq from same tissue type.

Supplemental Figure 7. High concordance of chromatin accessibility between peaks identified in two replicates. Peaks were identified in two replicates of liver (left) and testis (right). Log_{10}(P-Value) of each shared peak is highly correlated between replicates. Irreproducible Discovery Rate was calculated by calculated the ranked significance of reproducible and irreproducible peaks between replicates. Peaks with an $IDR < 0.05$ are shown in red.

Supplemental Figure 8. Verification of ATAC-seq proximity to annotated genes. Three representative examples of ATAC-seq read depth enriched at ACRs called with MACS2. The most proximal genes were identified with homer and with a custom python script. Annotations for a subset of 100 X chromosome ACR were visually inspected with JBrowse, to confirm that the peak was accurately assigned to nearby genes. ATAC-seq read depth is shown in grey. Bed files for the summit of each ACR identified by MACS2 is shown below the Ensembl gene annotations.

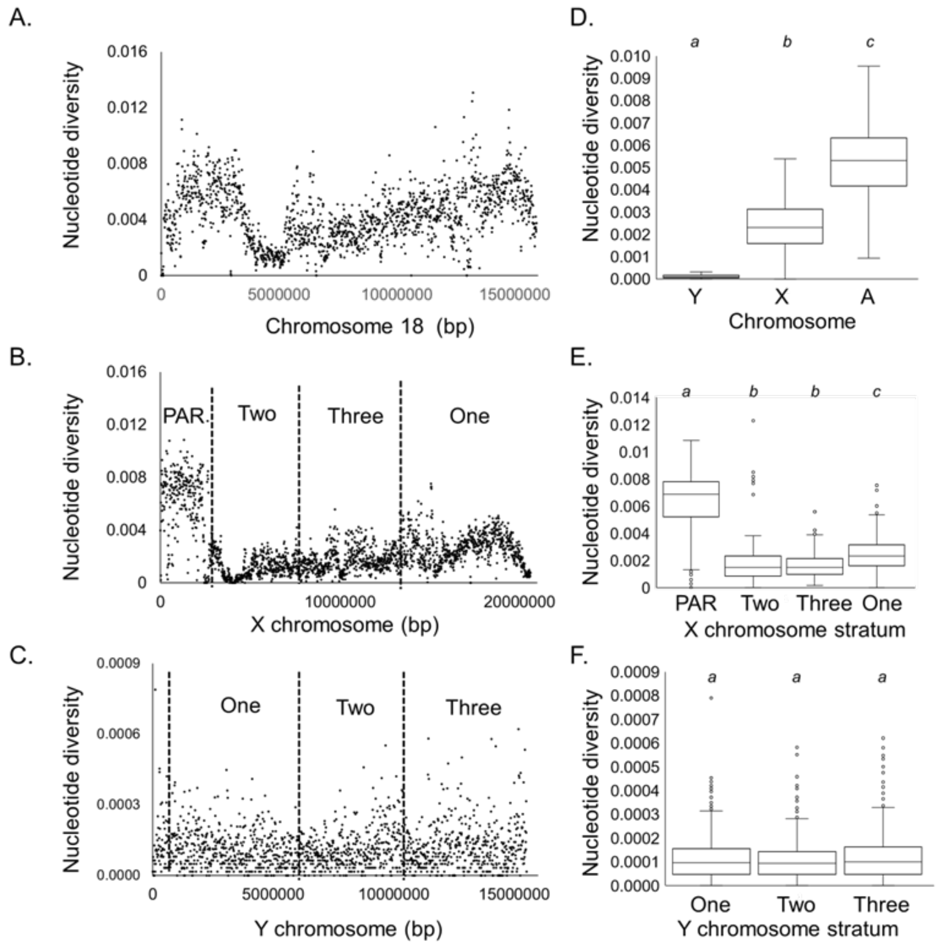

Supplemental Figure 1. Nucleotide diversity (pi) across the sex chromosomes and representative autosome.

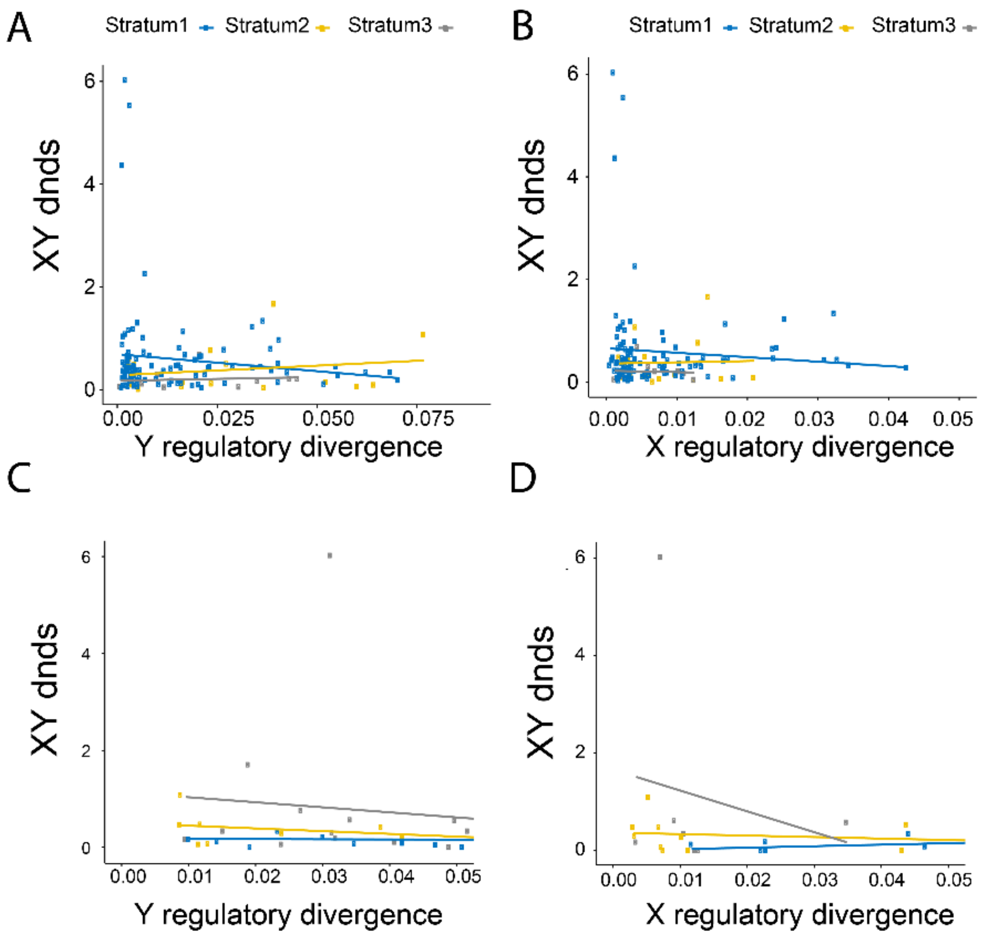

Supplemental Figure 2. d_N / d_S ratios of coding regions compared to ACR divergence.

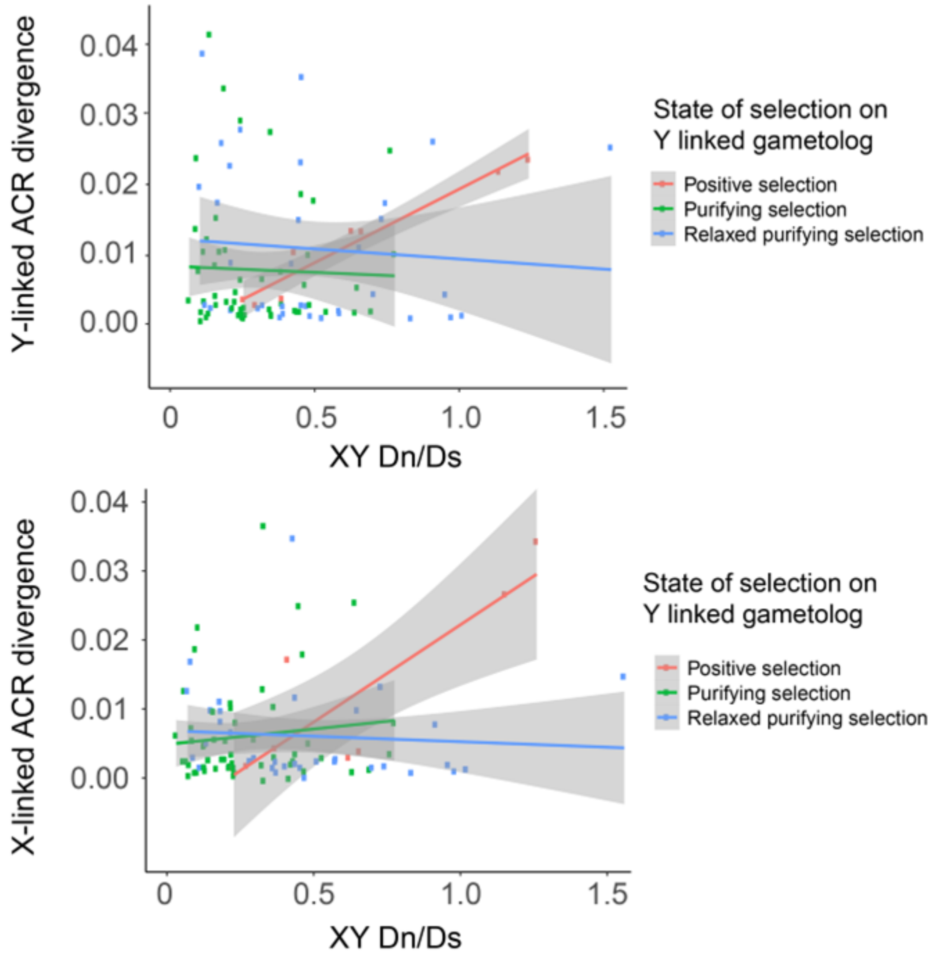

Supplemental Figure 3. Selective constraint of genes affects correlation between ACR and proximal coding divergence.

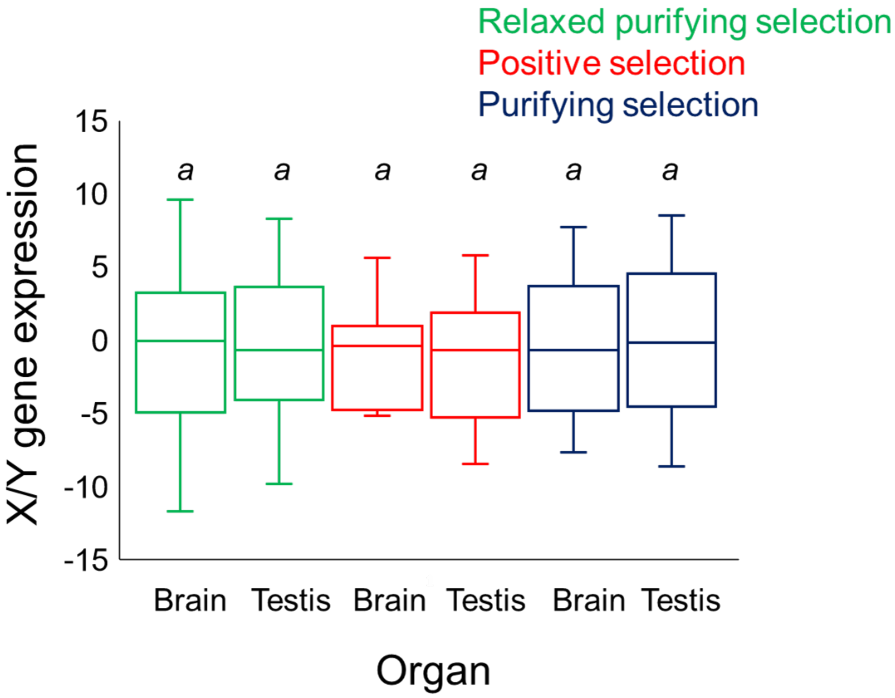

Supplemental figure 4. Allelic-specific expression for Y gametologs under different modes of selection

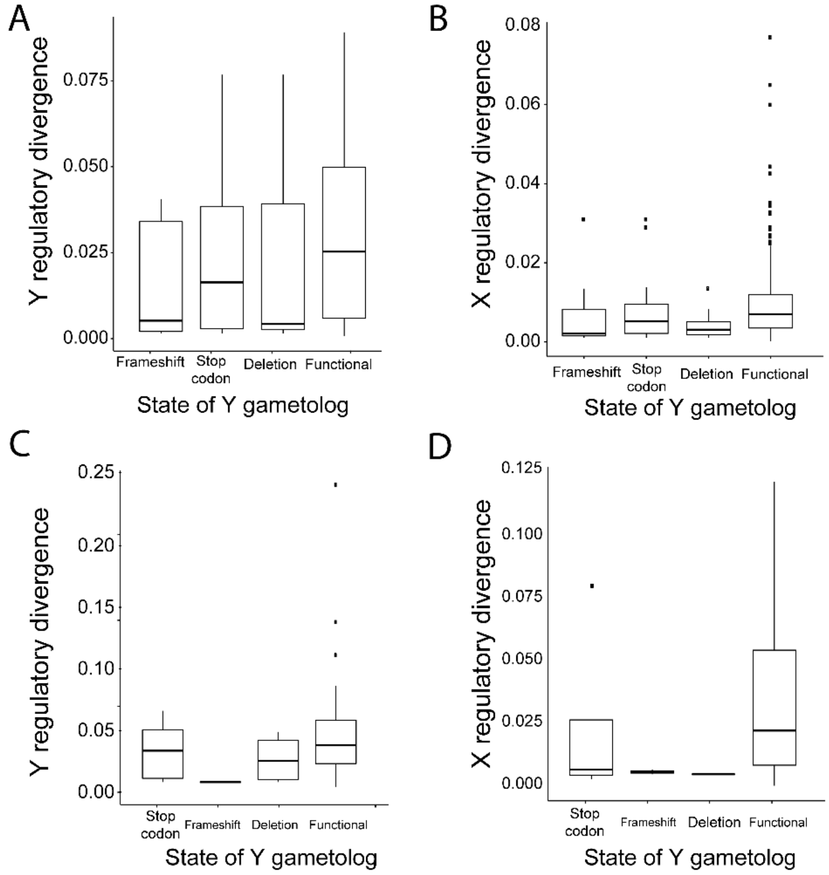

Supplemental Figure 5. ACR divergence compared to functional states of Y-linked gametologs.

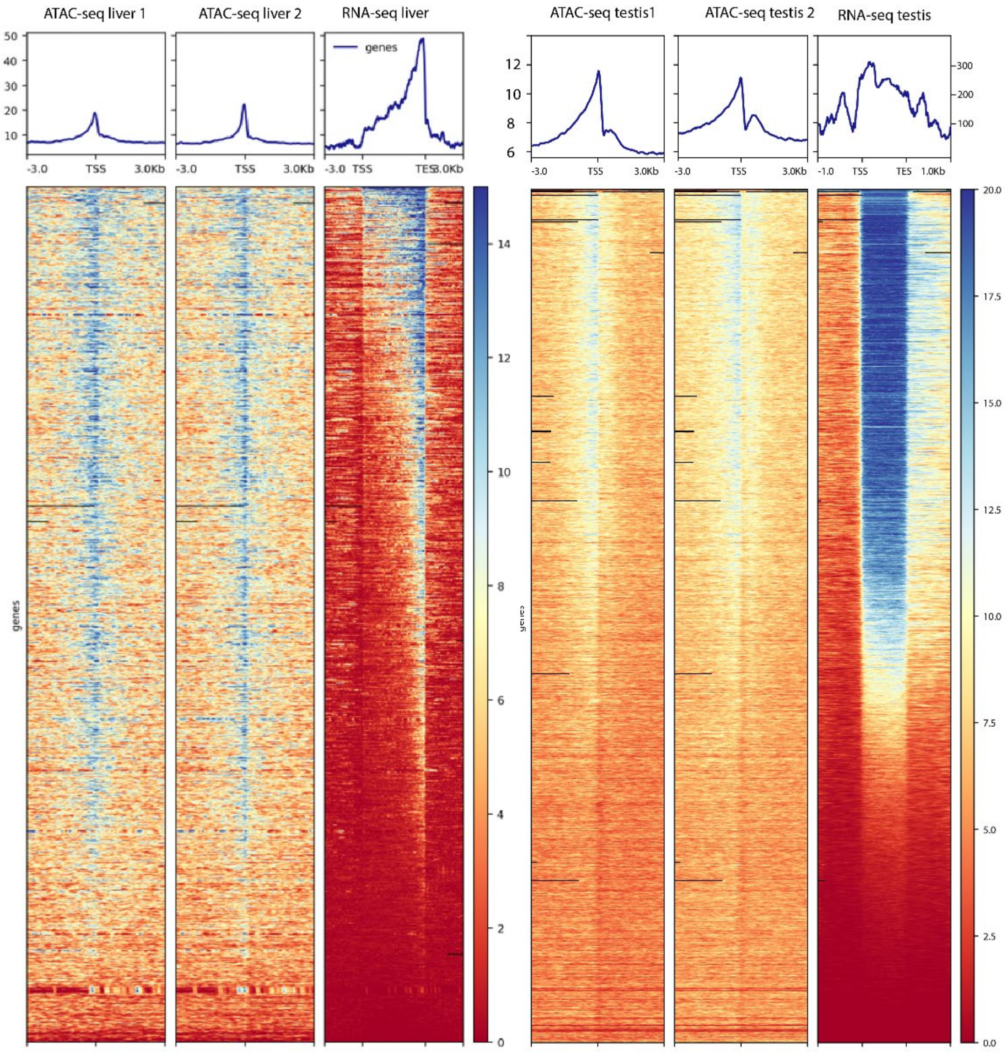

Supplemental Figure 6. High concordance of chromatin accessibility around transcription start site of expressed genes.

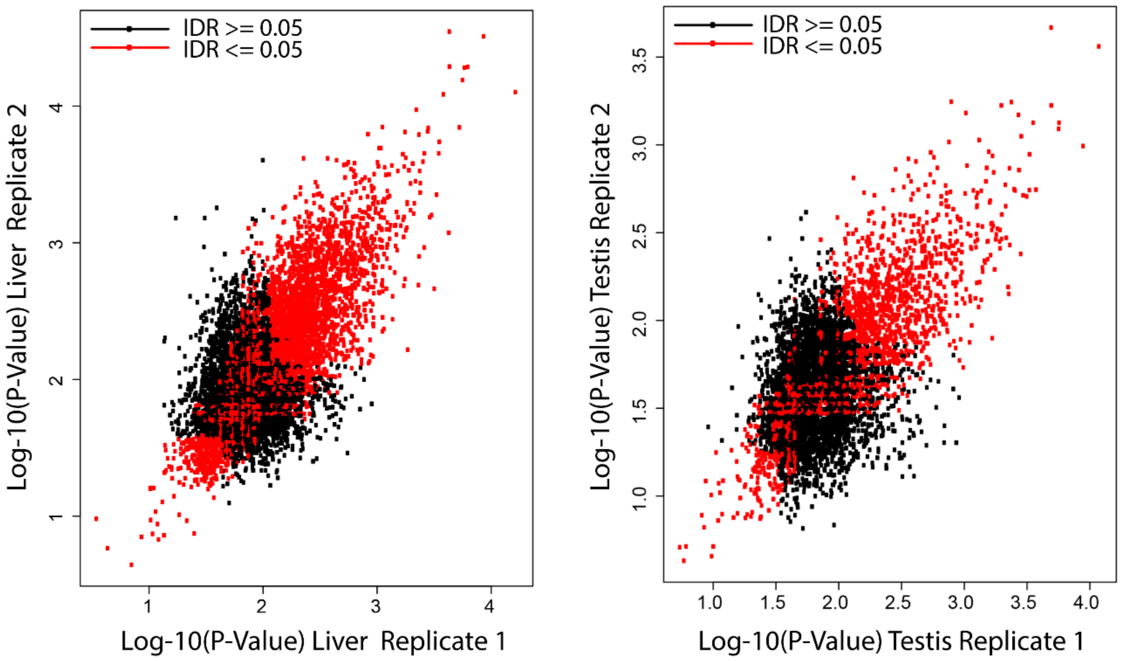

Supplemental Figure 7. High concordance of chromatin accessibility between peaks identified in two replicates.

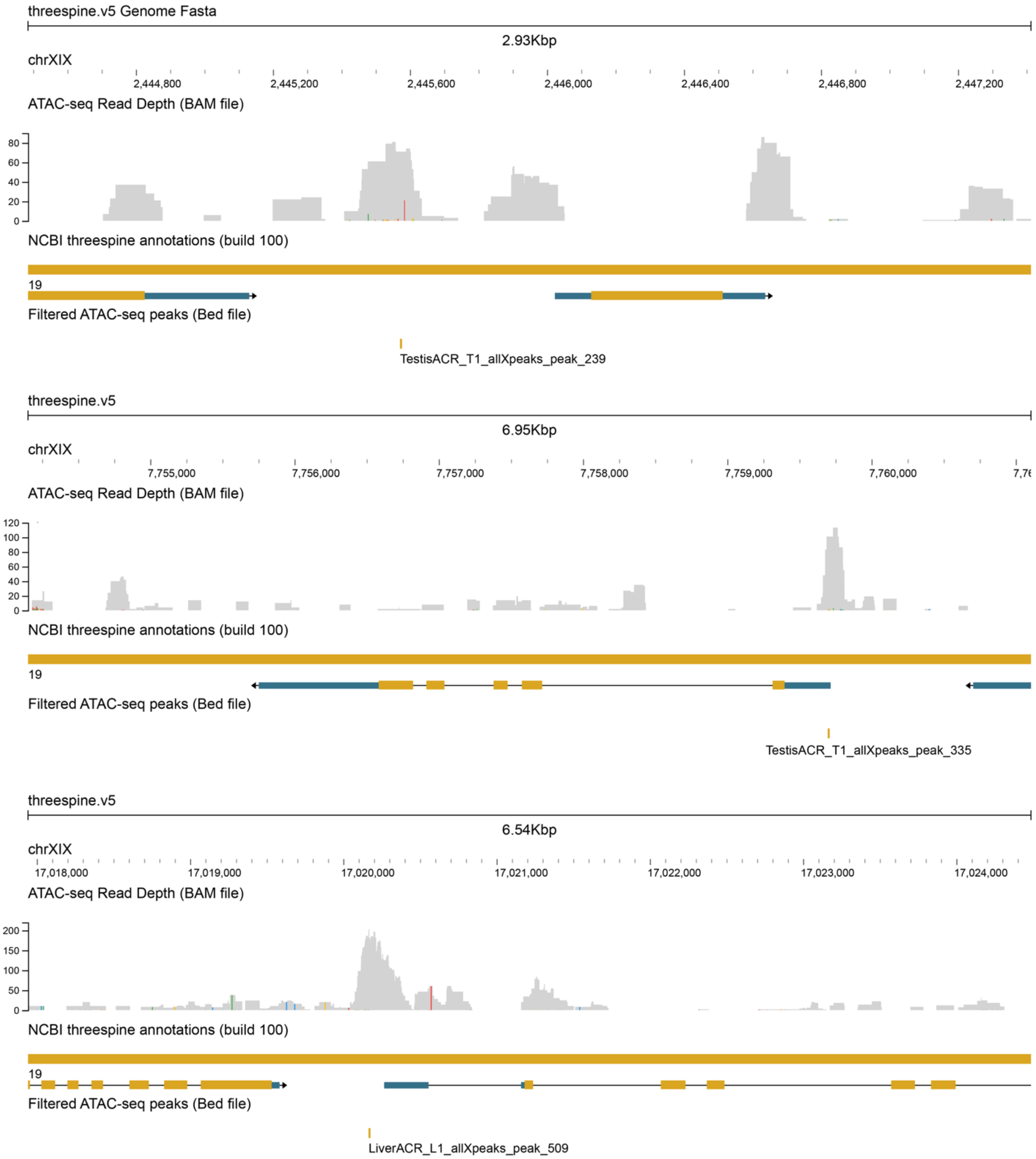

Supplemental Figure 8. Verification of ATAC-seq proximity to annotated genes.
